# Personality, Relationship Conflict, and Teamwork-Related Mental Models

**DOI:** 10.1371/journal.pone.0110223

**Published:** 2014-11-05

**Authors:** Delia Vîrgă, Petru Lucian CurŞeu, Laurenţiu Maricuţoiu, Florin A. Sava, Irina Macsinga, Silvia Măgurean

**Affiliations:** 1 Department of Psychology, West University of Timişoara, Timişoara, Romania; 2 Department of Organisation Studies, Tilburg University, Tilburg, The Netherlands; 3 Department of Psychology, “Babeş-Bolyai” University, Cluj-Napoca, Romania; University of Utah, United States of America

## Abstract

This study seeks to explore whether neuroticism, agreeableness, and conscientiousness moderate the influence of relationship conflict experienced in groups on changes in group members' evaluative cognitions related to teamwork quality (teamwork-related mental models). Data from 216 students, nested in 48 groups were analyzed using a multilevel modeling approach. Our results show that the experience of relationship conflict leads to a negative shift from the pre-task to the post-task teamwork-related mental models. Moreover, the results indicate that conscientiousness buffered the negative association between relationship conflict and the change in teamwork-related mental models. Our results did not support the hypothesized moderating effect of agreeableness and show that the detrimental effect of relationship conflict on the shift in teamwork-related mental models is accentuated for group members scoring low rather than high on neuroticism. These findings open new research venues for exploring the association between personality, coping styles and change in teamwork-related mental models.

## Introduction

Groups are multilevel systems in which the interplay between group members' attributes (e.g., personality traits, abilities, cognitions, and competencies) and factors pertaining to interpersonal interactions (e.g., group climate, teamwork quality) generates individual and group level outcomes and behaviors [1]–[3]. As part of a larger social system (the small group) individual members use evaluative cognitions to understand the workings and intricacies of these systems. These evaluative cognitions form their mental model related to teamwork [4]. Group members' engagement with the group task depends on the content of their teamwork-related mental models [4]; [5]. Because (1) the evaluative cognitions that group members hold in relation to teamwork are important drivers of task engagement and ultimately group performance [5] and (2) personality is one of the most explored predictors of work related attitudes and behaviors [6]; [7] it becomes important to identify individual differences (in particular personality traits) that influence the development and change of these individual mental models.

Research to date amply documented the association between group members' personality, on the one hand, and team performance [8]–[10] or teamwork quality [11], on the other hand. A core argument in this vein of research is that personality influences task and interpersonal engagement and ultimately group performance. Although intuitively appealed and abundantly supported by empirical evidence, this claim was often tested by looking at groups in a static manner. Personality does not only influence the engagement in social interactions, but personality traits also generate patterns of interpretation for the social interactions [12]. It is, therefore, meaningful to examine the way in which personality influences the “reading” of the dynamic group climate or in other words group members' individual mental models related to teamwork. Imagine, for example, how different group members perceive and relate to interpersonal frictions and relationship conflict. For a person scoring high on neuroticism, intense, and emotionally laden interpersonal conflicts bear a different significance than for a person scoring low on neuroticism. As such, one should expect personality driven differences in the way group members perceive the quality of interpersonal relations as they unfold in time.

Because relationship conflict reduces teamwork quality [1] and is an important interpersonal stressor associated with group work [13]; [14], we build on the differential exposure-reactivity model to argue that personality influences both the engagement in relationship conflict (stress exposure) as well as the coping strategies mobilized to deal with the stress triggered by it (reaction to stress). Therefore, personality is an important contingency of the teamwork mental model changes induced by the experience of relationship conflict in groups. These changes in the teamwork-related mental models (TWMM) reflect a dissonance reduction process, in which group members attempt to reach consistency between two different sets of evaluative cognitions: their original expectations towards teamwork and the evaluative cognitions referring to the teamwork quality experienced during real group work. The experience of relationship conflict increases this dissonance and conscientiousness, agreeableness and neuroticism are important contingencies in this dissonance reduction process as they influence the exposure and reactivity to interpersonal stressors.

Our conceptualization of the interplay between personality and relationship conflict is in line with the situational congruence model [15] and the person-environment fit framework [16]; [17] as we argue that change in teamwork mental models (the dissonance between expected teamwork quality and real teamwork quality experienced while performing the collective task) is less strong for situations of fit rather than a misfit between group members' personality and a conflicting group climate. In our study we are particularly interested in testing the way in which changes in the TWMM are influenced by the interaction between neuroticism, agreeableness, and conscientiousness, on the one hand, and perceptions of relationship conflict (as indicator of group climate), on the other hand.

### Teamwork-related mental models and relationship conflict

Working in groups raises important challenges for organizational members because they have to focus constantly on both interpersonal interactions and the task [18]. Interpersonal interactions in groups often involve conflicts [19] and relationship conflict is an important work stressor in modern organizations [13]; [14]. Therefore, relationship conflict reflects a negative group context marked by interpersonal frictions, negative emotionality, and task disengagement [14]; [19]. Meta-analytical findings converge in showing that relationship conflict is detrimental for task performance and satisfaction with the group [18]; [20] because it involves perceptions of threats to individual or group goals.

Moreover, Chen and colleagues [1] showed that relationship conflict reduces group members' psychological empowerment and their affective commitment to the group, which in turn decrease the likelihood of them engaging in teamwork behaviors. Imagine members of a newly formed group that hold rather positive expectations towards teamwork and they experience relationship conflict after they begin to work on the task. Their evaluative cognitions towards teamwork will become more negative, they will feel less psychologically empowered, less committed to the group and will eventually withdraw from the task. Therefore, the experience of relationship conflict creates discrepancies between the initial positive expectations towards teamwork and the quality of interpersonal interactions unfolding in reality. We argue, therefore, that the relationship conflict is an important teamwork related demand and it increases the cognitive discrepancy between the expected and realized teamwork quality. We, therefore, hypothesize that:

*Hypothesis 1: Relationship conflict leads to a negative shift in group members' teamwork-related mental models.*

### Person-environment fit in groups – reactions to relationship conflict

Person-environment fit models have been extensively used to document the way in which the fit between personality characteristics and various contextual variables at work influence work related behaviors and outcomes [17] and offers a valuable starting point for understanding the fit between personality traits and teamwork requirements. Research to date extensively explored the association between the big five personality dimensions and teamwork quality and performance [8]; [21]; [9]. In line with Mount and colleagues [21] and Peeters and colleagues [9], we argue that successful groups are composed of individuals with specific personality profiles that reflect both task engagement (conscientiousness) and interpersonal orientation (agreeableness). In their meta-analysis, Mount and colleagues [21] showed that conscientiousness, agreeableness, and emotional stability are positively related to performance in jobs involving interpersonal interactions. Their results also show that emotional stability and agreeableness have the strongest association with performance in jobs that involve teamwork. In other words, we could argue that people scoring high on conscientiousness, high on agreeableness, and low on neuroticism fit well with work contexts involving teamwork.

Working together with others in a group poses important demands on individual group members. They have to cope with the challenges associated with the collective task and also with the ones involved by frequent interpersonal interactions [13]. Therefore, in order to cope with the teamwork-related demands, group members need both task-related as well as interpersonal skills and competencies [22]. Although the literature to date explored a variety of proxies for teamwork related demands (ranging from task complexity to communication demands), relationship conflict is, by far, the most widely explored indicator, with both relational and performance correlates. Because it is related with reduced interpersonal satisfaction and decreased collective performance [20]; [18], we argue that relationship conflict is a comprehensive indicator of teamwork-related demands. In line with the person-environment fit framework, we focus here on a particular type of personality-demand fit, predicting that members with certain personality profiles cope better with the demands associated with relationship conflict and as such their teamwork mental models are less volatile over time.

In order to clarify the mechanisms at work in this person-environment fit framework, we use the differential exposure-reactivity model of personality and stress [23]. According to this model, group members' personality traits influence their likelihood of engaging in stressful situations (e.g., relationship conflict) as well as the selection of coping mechanisms they use to deal with the stressful consequences of relationship conflict. From the coping strategies summarized in the meta-analysis of Connor-Smith and Flachsbart [24], four are directly relevant for addressing the negative emotionality associated with relationship conflict in groups. The problem solving coping strategy involves attempts to reduce relationship conflict through careful task planning, keeping track of the progress towards the collective goals and staying engaged with the task. The emotional regulation focus involves the active attempts to reduce the incidence of negative emotions and the appropriate expression of emotional contents. The cognitive restructuring coping refers to ways in which relational frictions are cognitively reframed in order to reduce their negativity. Finally, the focus on negative emotions is an escalation coping strategy that increases the expression of negative emotions and ultimately leads to relationship conflict escalation. Table 1 summarizes the results reported in [24] concerning the association between conscientiousness, agreeableness, and neuroticism, on the one hand, and the above mentioned coping strategies, on the other hand.

**Table 1:** Conscientiousness, Aggreableness, Neuroticism and their relation with four coping strategies with interpersonal relevance.

Coping strategy	Conscientiousness	Agreableness	Neuroticism
**Problem solving**	Positive	Positive	Negative
**Emotion regulation**	Positive	Not significant	Not significant
**Cognitive restructuring**	Positive	Positive	Negative
**Negative emotion focus**	Negative	Negative	Positive

Note: The table presents a summary of the results reported in the meta-analysis by Connor-Smith and Flachsbart (2007).

We therefore build on the differential exposure-reactivity model of stress and personality to explore the way in which teamwork related mental models change in time as a function of experienced relationship conflict and we expect that conscientiousness, agreeableness, and neuroticism moderate the relationship between relationship conflict and changes in teamwork related mental models. Our main claim is that the three personality dimensions influence the exposure and reactivity to relationship conflict as an interpersonal stressor and as such they play an important role in reducing the cognitive dissonance between the expectations towards teamwork quality and the evaluative cognitions of real teamwork quality.

### Conscientiousness

Conscientious individuals are achievement oriented, orderly, punctual, dependable, self-disciplined and perceived by others as being task-oriented [12]. Group members scoring high on conscientiousness fit well with the task related demands in teamwork as they spend substantial effort on the task and engage in planning and organizing of group work [25]. Moreover, conscientious members fit well with the interpersonal demands and they perform well in activities that require interdependent and smooth interpersonal relationships, as they constantly help other members of the group to perform their tasks [26]; [27]. Using the terms of the differential exposure-reactivity model [23], conscientious group member tend to identify and avoid predictable interpersonal stressors [28], to preserve harmonious interpersonal relations and thus they are less likely to be exposed to the stress associated with relationship conflict.

When however, relationship conflict emerges in interpersonal situations, it generates frustration [14] and recent empirical evidence suggests that conscientious individuals are less likely to translate anger experienced as a consequence of interpersonally frustrating situations into aggression than individuals scoring low on conscientiousness [29]. Moreover, conscientiousness is positively correlated with effortful control [30] and given their high ability of suppressing a dominant response (anger and aggression) conscientious group members do not escalate relationship conflict in groups. Due to their high task orientation, their tendency to predict and avoid stressful interpersonal events as well as their effective strategies of modulating their dominant answers in interpersonal situations, we expect that conscientious group members will engage less in relationship conflict (low stress exposure) and will deploy effective coping strategies when relationship conflict occurs in their interpersonal relations (selection of effective coping strategies). To conclude, relational conflict is expected to deteriorate less the teamwork related mental models for those high rather than low on conscientiousness.

*Hypothesis 2: Conscientiousness buffers the association between relationship conflict and negative shift in teamwork-related mental models.*

### Agreeableness

Agreeable individuals are more likely to display open communication to be cooperative [31] than less agreeable individuals and display a caring orientation [25]. Also, agreeable people are motivated to establish and maintain good social relationships. Agreeable group members may facilitate the process of conflict resolution because they tend to be altruistic, compliant, and modest [31]; [32]. Moreover, agreeable people tend to maintain social harmony in the group and to reduce within-group competition [33] while group members scoring low on agreeableness could foster interpersonal conflict because they do not pay attention to the needs, concerns and general task related perspectives of the other group members [34]. Therefore, because agreeableness is the personality dimension with the strongest association with the quality of interpersonal relations, it is expected that group members scoring high on agreeableness fit well with the interpersonal demands associated with teamwork. In terms of exposure to relationship conflict, agreeable individuals tend to avoid conflicts and experience less interpersonal stress [12]; [28], while in terms of coping, agreeable group members tend to focus on problem solving strategies and cognitive restructuring [24] and by doing so they maintain a positive group climate. Agreeableness is also positively related to effortful cognitive [30] and emotional [35] control, and given these self-regulation reactions, we expect that agreeableness will buffer the change in TWMM associated with aversive relationship conflict.

*Hypothesis 3: Agreeableness buffers the association between relationship conflict and negative shift in teamwork-related mental models.*

### Neuroticism

Neuroticism or low level on emotional stability characterizes people who experience frustration, anxiety and depression that are usually associated with negative performance outcomes [25]. Neurotic individuals are also more sensitive to work-related stress and are less willing to help others [36]. Moreover, people scoring high on neuroticism tend to experience negative emotions [37]. As group members scoring high on neuroticism tend not to express their anger [12] and given that relationship conflict reflects emotionally laden situations, we argue that neuroticism is in misfit with relationship conflict demands. Neuroticism is also positively related with stress exposure and an ineffective selection of coping strategies [28]; [24] and group members scoring high on neuroticism are expected to experience a higher dissonance between their teamwork related expectations and the way the evaluate teamwork under high relationship conflict conditions. We therefore expect that group members scoring high on neuroticism tend to experience a significant drop in their positive perceptions of TWMM when relationship conflict increases. For members scoring low on neuroticism (emotionally stable group members) the decrease in the teamwork related mental models evaluation while experiencing relationship conflict is expected to be lower because they are more secure, calm, steady, and may engage stronger with teamwork [10] and can cope more effectively with the stress induced by relationships conflict [28]. Therefore we hypothesize that:

*Hypothesis 4: Neuroticism accentuates the association between relationship conflict and negative shift in teamwork-related mental models.*

## Method

### Ethics statement

The experimental procedures were approved by the Institutional Review Board at the West University of Timisoara, No 12675/2014. All participants gave their informed written consent to participate in the study.

### Participants and design

Participants were 216 students (83% women, with an average age of 20.75 years old) organized in 48 groups having 3 to 6 members. They were asked to participate in a creative group exercise. Their task was to use six drinking straws of equal sizes, a one meter long plastic strip, a duct tape and a plastic bowl to build a device that would prevent a dropped egg from breaking. Participants were asked to fill out two questionnaires one before and one after the creative task. Before actively engaging in the exercise the participants were asked to fill out a personality questionnaire (NEO FFI, [32]) and a questionnaire evaluating their teamwork quality expectations [4]. After finishing the task, the participants were asked to fill out the same questionnaire used to evaluate their teamwork related mental models, only this time the items referred to how they perceived teamwork quality in their own group and the amount of relationship conflict experienced in their group.

### Measures

*The big five personality dimensions* were evaluated with NEO Five Factor Inventory (NEO-FFI; [32]). This is a questionnaire that assesses each factor with 12 items rated on a five-point Likert scale. We used the Romanian version of the NEO-FFI by [38], who reported a satisfactory level of internal consistency (around.90 for all factors) and strong empirical support for the validity of the scales.

*Teamwork-related mental models* (TWMM) were evaluated using an individual teamwork expectations measure (pre-task) developed by Eby and colleagues [4] consisting of 28 items. Prior to the task engagement, participants were asked to rate their expectation concerning the way they will work together in the group (pre-task TWMM) and examples of items include: “Members share information with each other”. Answers were recorded on a 5 points Likert scale (1 =  *strongly disagree* to 5 =  *strongly agree*) and α = .91 for our sample. After finishing the creative task, each participant was asked to fill out the same items, but this time reflecting the way they actually worked together on the task (post-task TWMM). This scale has the same structure as the teamwork expectations scale but items were reframed to evaluate actual teamwork quality, e.g. “Members shared information with each other” (28 items; α = .88 post-task for our sample). The change in teamwork related mental models (TWMM) is calculated as the difference between TWMM assessment in the post-task and TWMM expectations in the pre-task.

*Relationship conflict* (RC) was assessed using a four item scale developed by Jehn [19]. Answers were recorded on a 5 points Likert scale (1 = *never* to 5 = *very often*) and examples of items include: “How often did you experience personality clashes between group members while working on the task?”. The internal consistency of the relationship conflict measure was.73 for our sample.

The *interpersonal acquaintance* level between group members (Acq) was assessed by asking each participant to rate how well he/she is acquainted with the other group members, using a 1 to 10 Likert scale (1 not at all –10 very well). For each participant we averaged the score of all evaluations to obtain an average acquaintance level of each individual with the rest of the group members. The Acq was used to control for any effects that might be attributed to this variable.

### Analyses

Given the nested nature of our data, we used a multilevel modeling approach. This decision is supported by the fact that the criterion variable (TWMM) is dependent both on individual characteristics and on the particular experience shared by all members of each specific group. Therefore we analyzed the data using a two-level model, in order to account for the non-independence of observations (the data sets used for the two levels are presented: level 1 data set is labeled “Data S1” and level 2 data set is labeled “Data S2”). At the individual level, we estimated intercept-only regression equations. These intercepts were used at the group level, for the prediction of TWMM change. Because we expected level-1 intercepts to vary randomly from one group to another, we conducted a group-level *random-intercept analysis*. For the multilevel analyses we used the hierarchical linear modeling (HLM) framework and performed our analyses with HLM 7 [39].

## Results

Means, standard deviation and correlations for the variables considered in the study are presented in Table 2. In order to simplify the interpretation of results and to reduce multicollinearity, all predictors were grand-mean centered before further analyses. We controlled for the level of interpersonal acquaintance in order to exclude the possibility that previous interpersonal interactions influence the individual expectations toward teamwork and biased the estimation of TWMM change. Interpersonal acquaintance was group mean centered before the analyses. For identification of moderation effects, we computed the cross-product vector of the predictors (relationship conflict with conscientiousness, agreeableness and neuroticism). Then, we included these three interaction terms as predictors into the level-1 equation. At the level 2 of the analysis, we expected that changes in TWMM will vary randomly from one group to another; therefore we assumed a random intercept model (Equation 2). However, we assumed that the relationships between predictors and the criterion will remain the same from one group to another, therefore we assumed a fixed slopes model (Equations 3 to 10).

**Table 2:** Means, standard deviations and correlations between variables at individual level.

Variables	Mean	SD	1	2	3	4	5	6	7	8	9
1. Age	20.75	3.45	1								
2. Gender	.84	.36	−.07	1							
3. Interpersonal acquaintance	5.40	2.49	−.16^*^	.06	1						
4. Centered Neuroticism	0	7.44	−.11	.05	.07	1					
5. Centered Agreeableness	0	5.33	.06	−.01	.06	−.19^**^	1				
6. Centered Conscientiousness	0	6.64	.03	.04	.03	−.23^**^	.18^**^	1			
7. Centered relationship conflict	0	.41	−.06	−.12	.04	.11	−.07	−.13	1		
8. TWMM pre-task	3.99	.37	−.02	.03	−.01	.13	.03	−.07	.04	1	
9. TWMM post-task	3.96	.45	.09	.06	.11	−.04	.07	.11	−.25^**^	−.02	1
10. TWMM change	−.02	.59	.08	.02	.09	−.11	.03	.13^*^	−.22^**^	−.65^**^	.77^**^

Notes:* Correlation is significant at the 0.05 level (2-tailed)**; Correlation is significant at the 0.01 level (2-tailed); scores for the four main predictors were centered; TWMM – teamwork-related mental model; TWMM change reflects the difference in teamwork-related mental models in post-task as compared to the teamwork-related expectations in the pre-task conditions (TWMM post task minus TWMM pre-task); gender is coded as dummy variable with 1 = women and 0 = men.

(N = 216).

Level 1 equation
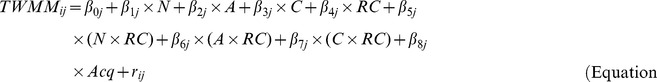
(Equation \;1)


Level 2 equations
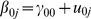
(Equation 2)

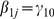
(Equation 3)

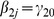
(Equation 4)

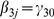
(Equation 5)

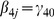
(Equation 6)

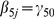
(Equation 7)

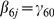
(Equation 8)

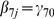
(Equation 9)

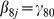
(Equation 10)


### Correlations between study variables, at individual level

Results presented in Table 2 indicated significant correlations between change in TWMM and conscientiousness (*r* = 0.13, *p*<.05), on the one hand, and relationship conflict (RC) (*r* = −0.22, *p*<.01), on the other hand. The correlations between the change in TWMM and Neuroticism were marginally significant (*r* = −0.11, *p* = .08), and not significant with Agreeableness (r = 0.03, p>.05). Although these two variables were not significantly associated with change in TWMM, we included them into further analyses to investigate whether they act as moderators between RC and change in TWMM.

### The null model

In the first step of the multilevel analysis, we examined variance components using the null model (equations for both levels with only intercepts), in order to see how the variance of perceived TWMM change is partitioned between the two levels of analysis. Results indicated that 17.9% of the change in TWMM variance lies between groups and the intercepts vary significantly between groups (Wald Z = 2.34, *p*<.05). Taken together, these results indicated that a multilevel approach is adequate for the analysis of our data.

### The group level random intercept model

The results of the multilevel analysis are presented in Table 3. Regarding the direct effects, RC was a significant predictor of change in TWMM (γ_40_  = −0.28, *t* = −2.39, *p*<.05), indicating that individuals in groups that experienced high RC significantly decreased their post-task evaluation of the TWMM, as compared with their pre-task expectation of TWMM. Therefore Hypothesis 1 is fully supported. The relations between personality variables and change in TWMM were significant in the case of Conscientiousness (γ_30_ = 0.01, *t* = 2.33, *p*<.05), and not significant in the case of Neuroticism and Agreeableness. The level of between-participants acquaintance had little impact on the overall results, because (a) it was not correlated with any of the variables included in the analysis, and (b) it did not predict significantly the change in TWMM.

**Table 3:** Multilevel results for the prediction of TWMM change.

Parameter	Estimate	Std. Error	df	t	p
Intercept (γ_00_)	−.024	.049	47	−.49	.63
N (γ_10_)	−.005	.005	160	−.97	.33
A (γ_20_)	−.001	.007	160	−.11	.92
C (γ_30_)	.012	.005	160	2.33	.02
RC (γ_40_)	−.280	.117	160	−2.39	.02
RC x N (γ_50_)	.031	.009	160	3.23	.002
RC x A (γ_60_)	−.025	.015	160	−1.58	.12
RC x C (γ_70_)	.036	.014	160	2.60	.01
Acq	.003	.019	160	.20	.84

Notes: N– neuroticism; A– agreeableness; C– conscientiousness; RC – relationship conflict; Acq – interpersonal acquaintance.

**Figure 1:**
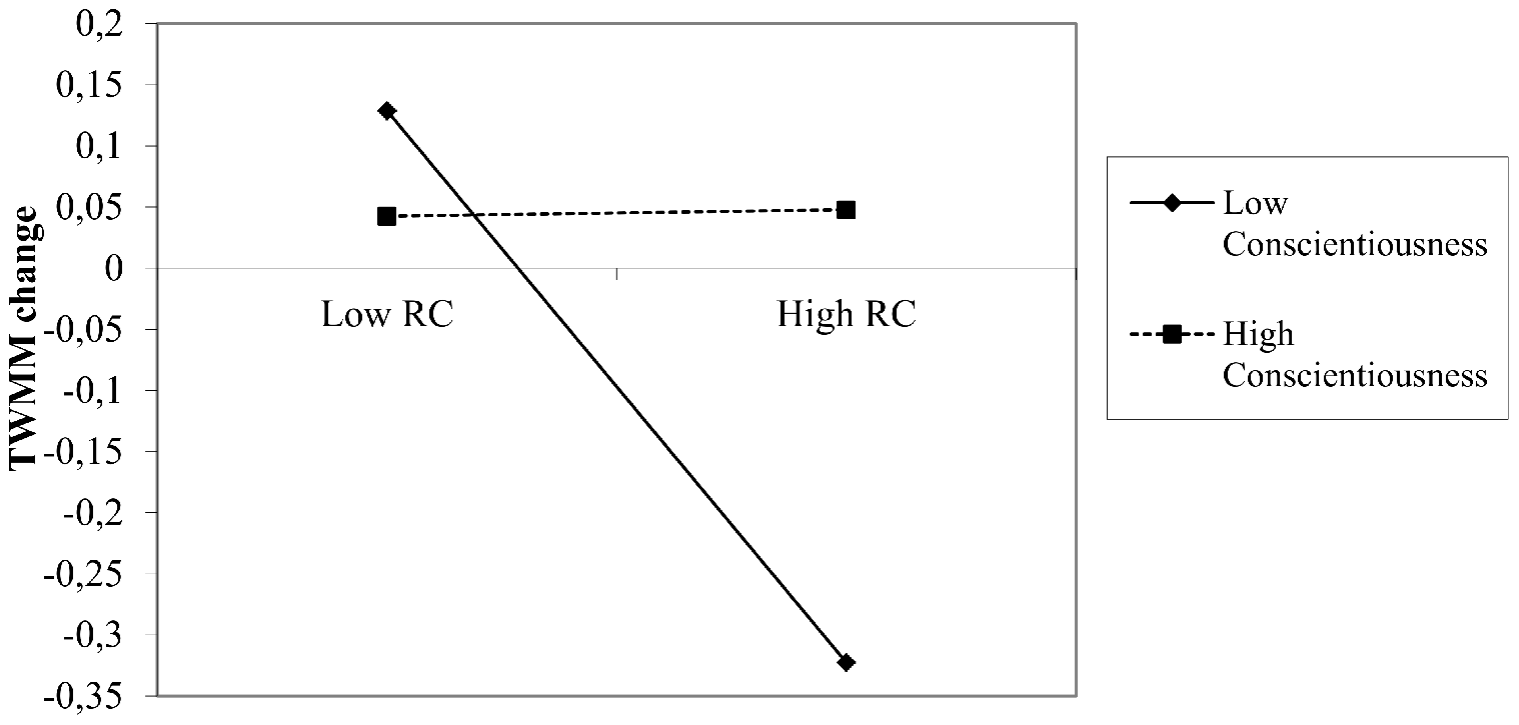
The Moderation Effect of Conscientiousness on the Relation between Relationship Conflict and TWMM Change. Note: TWMM change reflects the difference in teamwork-related mental models (TWMM post-task minus TWMM pre-task).

As the interaction between agreeableness and RC is not significant, we can conclude that Hypothesis 3 is not supported. The multilevel analysis indicated the presence of two significant moderation effects. First, the interaction term between conscientiousness and RC was statistically significant (γ_70_ = 0.036, *t* = 2.60, *p*<.05). This moderation effect is presented in Figure 1, and indicated that a negative association between RC and change in TWMM can be found only in the case of group members scoring low on conscientiousness, but not in the case of individuals scoring high on conscientiousness. Therefore, Hypothesis 2 was fully supported by the data.

**Figure 2:**
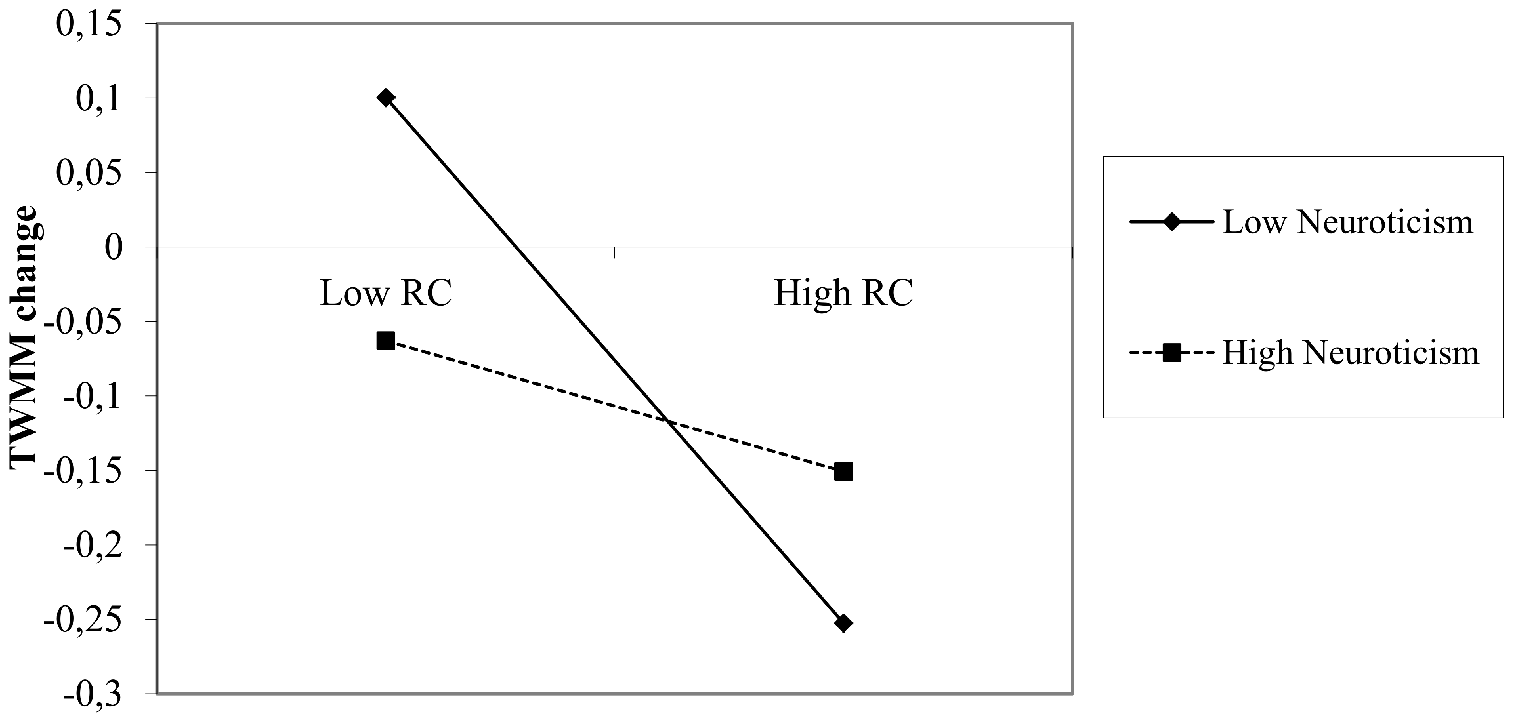
The Moderation Effect of Neuroticism on the Relation between Relationship Conflict and TWMM Change. Note: TWMM change reflects the difference in teamwork-related mental models (TWMM post-task minus TWMM pre-task).

Second, the cross-product term between neuroticism and RC was statistically significant (γ_50_ = 0.03, *t* = 3.23, *p*<.01), indicating that the association between RC and change in TWMM is also moderated by neuroticism. This moderation effect is presented in Figure 2, and showed that a negative correlation between RC and change in TWMM can be found only in the case of group members with low levels of neuroticism, and not in the case of group members with high levels of neuroticism. Although the interaction effect is significant, it reveals a different effect of the interplay between neuroticism and RC than expected in Hypothesis 4. The slopes of this interaction are presented in Figure 2.

## Discussion

This study used a multilevel perspective on groups to test the extent to which personality moderates the deteriorating effect of relationship conflict on change in TWMM. As expected our results show that the experience of relationship conflict deteriorates TWMM supporting the claim that intra-group conflict is an important stressor in groups [13]; [40]. Building on a person-environment fit perspective we argued that high conscientiousness, high agreeableness, and low neuroticism reflect a good fit between the group members and the teamwork related demands as captured by relationship conflict. We argued that when group members experience relationship conflict, high conscientiousness, high agreeableness and low neuroticism facilitate the dissonance reduction process responsible for attenuating the discrepancy between TWMM in the pre-task and post task conditions. Our results support the claim that conscientiousness is a buffer that reduces the detrimental effects of relationship conflict on TWMM and shows that relationship conflict decreases the favorable evaluations of teamwork quality only for those group members scoring low on conscientiousness. This result is in line with previous findings showing that the personality has the potential to affect employees' perceptions and appraisals of the work related environment, their causal attributions for work related events [41]. In line with the attentional-resource perspective, we argue that high relationship conflict alters attributions that group members make about each others' actions and behaviors during teamwork [42]. When experiencing relationship conflict, group members scoring low on conscientiousness will most probably translate anger into aggression, withdraw from the task and their disengagement and aggression decreases the quality of teamwork interactions and consequently their perceptions of teamwork quality will deteriorate in the post-task condition. Conscientious group members, on the other hand, are more likely to tackle interpersonal issues associated with relationship conflict, stay focused and help others focus on the task. Moreover, conscientious group members have a high capacity for cognitive control and they are effective in cognitive restructuring attempts [28] therefore they report almost no shift in teamwork related mental models attributable to relationship conflict.

Our results did not support the moderating role of agreeableness. The key argument was that agreeableness influences the process of dissonance reduction in the TWMM change through the engagement with relationship conflict as an interpersonal stressor and the selection of effective coping strategies. This argument is in line with the claim that the interpersonal processes associated with agreeableness are the result of cognitive self-regulation mechanisms and not just with conformity and social desirability [29]. A possible explanation for the lack of support for Hypothesis 3 can be that the student group evaluated in this research worked together for a short period of time. As the groups were in principle formed for just one task and the group members had no foreseeable future interactions, agreeableness apparently was less important for buffering the negative effect of relationship conflict on TWMM change. The degree of group permanency influences the use of emotion regulation strategies in dealing with conflict [43] and it is not unreasonable to argue that people scoring high on agreeableness will mobilize their emotion regulation and effortful control strategies only when they expect future interpersonal interactions with their group members. Agreeableness therefore did not influence the dissonance reduction process associated with the experience of relationship conflict in groups. A valuable direction for future research is to explore the way in which the degree of group permanency influences the relationship between agreeableness and cognitive change induced by relationship conflict. Future research could also attempt to disentangle the association between agreeableness and the deployment of response focused (short term perspective) versus antecedent focused (long term perspective) emotion regulation strategies triggered by the experience of relationship conflict.

With respect to neuroticism, we hypothesized that the association between relationship conflict and decrease in teamwork-related mental models is higher for emotionally unstable individuals. Opposed to our expectations, we found that group members scoring low on neuroticism report a negative association between relationship conflict and teamwork-related mental models. Emotional stability seems, therefore, not to play the buffering role we expected. One alternative explanation is that the group members scoring high on neuroticism are less likely to express their anger while being engaged in relationship conflict and this could prevent the further escalation of relationship conflict. Another alternative explanation refers to the tendency of people scoring high on neuroticism to adopt an emotion focused coping strategy [44] that will eventually help them to cope with the negative emotionality associated with relationship conflict [14]. This would explain why for people scoring high on neuroticism the change in the teamwork related mental model is as strongly influenced by the relationship conflict as for people scoring low on neuroticism. The teamwork related mental model for people scoring high on neuroticism seems to fall below their original expectations independent of the level of relationship conflict experienced (see the regression slope in Figure 2). For emotionally stable group members, the post-task teamwork mental model exceeds their pre-task expectations when they experience less rather than more relationship conflict. An explanation could be their involvement in the conflict and as they engage in relational frictions with others they lose their task focus, and as a consequence they report that their teamwork quality expectations are not met by the real group interactions. This counter-intuitive result most certainly warrants some further exploration and the way in which neuroticism relates to relationship conflict and conflict management in groups further extend our understanding of the interplay between personality and conflict in groups.

### Limitations and future research directions

Several limitations of the present study should be noted. First, we have to acknowledge the low number of groups with a short life span. Also, our study is limited in its ecological validity because the activity of student groups was performed in laboratory, not in real life environment. Thus, a replication of our study in established work group could further examine if agreeableness buffers the negative effects of relationship conflict on teamwork mental models. The relationship conflict and teamwork mental models measures were collected from group members using a self-report questionnaire. Separating the evaluations in time helped us to reduce the problems associated with common method variance. In terms of practical implications, our results show that conscientious group members successfully cope with relationship conflicts without altering their teamwork related mental models. Thus, conscientiousness is not only a good predictor of job performance, but it is also a good predictor for the adaptability of individuals in work groups. Further research should more directly address the plausible link between personality, preferred coping style, and relationship conflict. As literature to date started to explore the association between preferred coping styles in groups and relationship conflict [14], future research could extend this stream of literature by testing the extent to which coping styles mediate the association between personality dimensions and conflict escalation and transformation in groups.

## Supporting Information

Data S1 Original data files for the individual level of analysis.
(SAV)

Data S2 Original data files for the group level of analysis.
(SAV)

## References

[1] Chen G, Sharma PN, Edinger SK, Shapiro DL, Far JL (2011) Motivating and demotivating forces in teams: cross-level influences of empowering leadership and relationship conflict. J Appl Psychol 96: 541–557. 21171730 10.1037/a0021886

[2] Kozlowski SWJ (2012) Groups and teams in organizations: Studying the multilevel dynamics of emergence. In: Hollingshead AB, Poole MS, editors.Research methods for studying groups and teams: A guide to approaches, tools, and technologies. New York: Routledge.pp. 260–283.

[3] Kozlowski SWJ, Chao GT, Grand JA, Braun MT, Kuljanin G (2013) Advancing multilevel research design: Capturing the dynamics of emergence. Organ Res Methods 16: 581–615.

[4] Eby LT, Meade AW, Parisi AG, Douthitt SS (1999) The development of an individual-level teamwork expectations measure and the application of a within-group agreement statistic to assess shared expectations for teamwork, Organ Res Methods. 2: 366–394.

[5] Mathieu JE, Heffner TS, Goodwin GF, Salas E, Cannon-Bowers JA (2000) The influence of shared mental models on team process and performance. J Appl Psychol 85: 273–283. 10783543 10.1037/0021-9010.85.2.273

[6] Barrick MR, Mount MK, Judge TA (2001) Personality and performance at the beginning of the new millennium: What do we know and where do we go next? Int J Select Assess 9: 9–30.

[7] Judge TA, Ilies R (2002) Relationship of personality and performance motivation: A meta-analysis. J Appl Psychol 87: 797–807. 12184582 10.1037/0021-9010.87.4.797

[8] Arnulf JK (2012) Organizational change capacity and composition of management teams: A visualization of how personality traits may restrain team adaptability. Team Perform Manage 18: 433–454.

[9] Peeters MAG, Van Tuijl HFJM, Rutte CG, Reymen IMMJ (2006) Personality and team performance: A meta-analysis. Eur J Pers 20: 377–396.

[10] Bell ST (2007) Deep-level composition variables as predictors of team performance: A meta-analysis. J Appl Psychol 82: 62–78. 10.1037/0021-9010.92.3.595 17484544

[11] Driskell JE, Goodwing GV, Salas E, O′Shea PG (2006) What makes a good team player? Personality and team effectiveness. Group Dyn-Theor Res 10: 249–271.

[12] Bono JE, Boles TL, Judge TA, Lauver KJ (2002) The role of personality in task and relationship conflict. J Pers 70: 311–344. 12049163 10.1111/1467-6494.05007

[13] Ilies R, Johnson MD, Judge TA, Keeney J (2011) A within-individual study of interpersonal conflict as a work stressor: Dispositional and situational moderators. J Organ Behav 32: 44–64.

[14] Pluut H, CurŞeu PL (2013) Perceptions of intragroup conflict: The effect of coping strategies on conflict transformation and escalation. Group Process Intergroup Relat 16: 412–425.

[15] Pervin LA (1968) Performance and satisfaction as a function of individual-environment fit. Psychol Bull 69: 56–68.

[16] Caplan RD (1987) Person-environment fit theory and organizations: Commensurate dimensions, time perspectives and mechanisms. J Vocat Behav 31: 248–267.

[17] Edwards JR (2008) Person-environment fit in organizations: An assessment of theoretical progress. Acad Manag Ann 2: 167–230.

[18] De Dreu CKW, Weingart LR (2003) Task versus relationship conflict, team performance, and team member satisfaction: A meta-analysis. J Appl Psychol 88: 741–749. 12940412 10.1037/0021-9010.88.4.741

[19] Jehn KA (1995) A multimethod examination examination of the benefits and detriments of intragroup conflict. Adm Sci Q 40: 256–282.

[20] De Wit FRC, Greer LL, Jehn KA (2012) The paradox of intragroup conflict: A meta-analysis. J Appl Psychol 97: 360–390. 21842974 10.1037/a0024844

[21] Mount MK, Barrick MR, Stewart GL (1998) Five-Factor model of personality and performance in jobs involving interpersonal interactions. Hum Perform 11: 145–165.

[22] Stevens MJ, Campion MA (1994) The knowledge, skill and ability requirements for teamwork: Implications for human resource management. J Manage 20: 503–530.

[23] Bolger N, Zuckerman A (1995) A framework for studying personality in the stress process. J Pers Soc Psychol 69: 890–902. 7473036 10.1037//0022-3514.69.5.890

[24] Connor-Smith JK, Flachsbart C (2007) Relationships between personality and coping: A meta-analysis. J Pers Soc Psychol 93: 1080–1107. 18072856 10.1037/0022-3514.93.6.1080

[25] Prewett MS, Walvoord AAG, Stilson FRB, Rossi ME, Brannick MT (2009) The team personality–team performance relationship revisited: The impact of criterion choice, pattern of workflow, and method of aggregation. Hum Perform 22: 273–296.

[26] LePine JA, Van Dyne LV (2001) Voice and cooperative behavior as contrasting forms of contextual performance: Evidence of differential relationships with Big Five personality characteristics and cognitive ability. J Appl Psychol 86: 326–336. 11393444 10.1037/0021-9010.86.2.326

[27] Porter KOLH, Hollenbeck JR, Ilgen DR, Ellis APJ, West BJ, et al (2003) Backing up behaviors in teams: The role of personality and legitimacy of need. J Appl Psychol 88: 391–403. 12814289 10.1037/0021-9010.88.3.391

[28] Carver CS, Connor-Smith JK (2010) Personality and coping, Annu Rev Psychol. 61: 679–704. 10.1146/annurev.psych.093008.100352 19572784

[29] Jensen-Campbell LA, Knack JM, Waldrip AM, Campbell SD (2007) Do Big Five personality traits associated with self-control influence the regulation of anger and aggression? J Res Pers, 41, 403–424.

[30] Jensen-Campbell LA, Rosselli M, Workman KA, Santisi M, Rios JD, et al (2002) Agreeableness, conscientiousness, and effortful control processes. J Res Pers 36: 476–489.

[31] Neuman GA, Wright J (1999) Team effectiveness: Beyond skills and cognitive ability. J Appl Psychol 84: 376–389. 10380418 10.1037/0021-9010.84.3.376

[32] Costa PT, McCrae RR (1992) Revised NEO Personality Inventory (NEO-PI-R) and NEO Five Factor (NEO-FFI) inventory professional manual. Odessa, FL: Psychological Assessment Resources, Inc. 101 p.

[33] Graziano WG, Hair EC, Finch JF (1997) Competitiveness mediates the link between personality and group performance. J Pers Soc Psychol 73: 1394–1408. 9418284 10.1037//0022-3514.73.6.1394

[34] Lord W (2007)NEO PI-R: A guide for interpretation and feedback in the work context, Oxford: Hogrefe Ltd. 99 p.

[35] Tobin RM, Graziano WG, Vanman EJ, Tassinary LG (2000) Personality, emotional experience, and efforts to control emotions. J Pers Soc Psychol 79: 656–669. 11045745

[36] Le H, Oh I, Robbins SB, Ilies R, Holland E, et al (2011) Too much of a good thing: Curvilinear relationships between personality traits and job performance. J Appl Psychol 96: 113–133. 20939656 10.1037/a0021016

[37] Langelaan S, Bakker AB, Van Doornen LJP, Schaufeli WB (2006) Burnout and work engagement: Do individual differences make a difference? Pers Individ Dif 40: 521–532.

[38] Iliescu D, Minulescu M, Nedelcea C, Ispas D (2009) NEO PI-R: Manual tehnic si interpretativ (Romanian version, adapted from Costa, P.T., McCrae, R. R, )Bucuresti: Sinapsis. 260 p.

[39] Raudenbush SW, Bryk AS, Congdon R (2010) HLM 7: Hierarchical linear and nonlinear modeling. Lincolnwood, IL: Scientific Software International.

[40] Peterson RS, Behfar KJ (2003) The dynamic relationship between performance feedback, trust, and conflict in groups: A longitudinal study. Organ Behav Hum Decis Process 92: 102–112.

[41] Spector PE (2011) The relationship of personality to counterproductive work behavior (CWB): An integration of perspectives. Human Resource Management Review, 21: , 342–352.

[42] Shaw JD, Zhu J, Duffy MK, Scott KL, Shih HA, et al (2011) A contingency model of conflict and team effectiveness. J Appl Psychol 96: 391–400. 20939655 10.1037/a0021340

[43] CurŞeu PL, BoroŞ S, Oerlemans LA (2012) Task and relationship conflict in short-term and long-term groups: The critical role of emotion regulation. Int J Conf Manag 23: 97–107.

[44] Brebner J (2001) Personality and stress coping. Pers Individ Dif 31: 317–327.

