# Correction: Personality, Relationship Conflict, and Teamwork-Related Mental Models

**DOI:** 10.1371/journal.pone.0116425

**Published:** 2014-12-23

**Authors:** The PLOS ONE Staff

The second author's name displays incorrectly. The correct name is: Petru Lucian Curşeu. The correct citation is: Vîrgă D, Curşeu PL, Maricuţoiu L, Sava FA, Macsinga I, et al. (2014) Personality, Relationship Conflict, and Teamwork-Related Mental Models. PLoS ONE 9(11): e110223. doi:10.1371/journal.pone.0110223
